# Artificial Light at Night Increases Recruitment of New Neurons and Differentially Affects Various Brain Regions in Female Zebra Finches

**DOI:** 10.3390/ijms21176140

**Published:** 2020-08-26

**Authors:** Stan Moaraf, Rachel Heiblum, Yulia Vistoropsky, Monika Okuliarová, Michal Zeman, Anat Barnea

**Affiliations:** 1 School of Zoology, Tel-Aviv University, Tel-Aviv 6997801, Israel; 2 Department of Natural and Life Sciences, The Open University of Israel, Ra’anana 43710, Israel; rheiblum@weizmann.ac.il (R.H.); uliakatz@hotmail.com (Y.V.); anatba@openu.ac.il (A.B.); 3 Department of Animal Physiology and Ethology, Faculty of Natural Sciences, Comenius University, 84215 Bratislava, Slovak Republic; monika.okuliarova@uniba.sk (M.O.); michal.zeman@uniba.sk (M.Z.)

**Keywords:** artificial light at night (ALAN), brain plasticity, new neuronal recruitment, neuronal densities, hippocampus (HC), Medial striatum (MSt), Nidopallium caudale (NC), melatonin, birds, zebra finches (*Taeniopygia guttata*)

## Abstract

Despite growing evidence that demonstrate adverse effects of artificial light at night (ALAN) on many species, relatively little is known regarding its effects on brain plasticity in birds. We recently showed that although ALAN increases cell proliferation in brains of birds, neuronal densities in two brain regions decreased, indicating neuronal death, which might be due to mortality of newly produced neurons or of existing ones. Therefore, in the present study we studied the effect of long-term ALAN on the recruitment of newborn neurons into their target regions in the brain. Accordingly, we exposed zebra finches (*Taeniopygia guttata*) to 5 lux ALAN, and analysed new neuronal recruitment and total neuronal densities in several brain regions. We found that ALAN increased neuronal recruitment, possibly as a compensatory response to ALAN-induced neuronal death, and/or due to increased nocturnal locomotor activity caused by sleep disruption. Moreover, ALAN also had a differential temporal effect on neuronal densities, because hippocampus was more sensitive to ALAN and its neuronal densities were more affected than in other brain regions. Nocturnal melatonin levels under ALAN were significantly lower compared to controls, indicating that very low ALAN intensities suppress melatonin not only in nocturnal, but also in diurnal species.

## 1. Introduction

Despite a growing number of studies that demonstrate adverse effects of artificial light at night (ALAN) on the behaviour and physiology of many species (e.g., reviewed in [1,2]), still, in birds relatively little is known regarding its effects on brain plasticity. The few existing studies report a decrease in the numbers of newly formed neurons in the hippocampus (HC) of Indian house crows (*Corvus splendens*) [3], and a decrease in soma size of neurons in the HC and the lateral caudal nidopallium (NCL) in this species, suggesting reduced neuronal plasticity [4]. We have also studied the effect of ALAN on neuronal plasticity in another diurnal species, zebra finches (*Taeniopygia guttata*), which have excellent visual abilities [5] and their physiology, reproduction and survival are greatly affected by circadian and circannual rhythms [6,7]. Our recent study [8] was the first to demonstrate that ALAN increases cell proliferation in brains of diurnal birds. More specifically, we found, in female zebra finches, that ecologically relevant intensities (0.5, 1.5, and 5 lux) of ALAN significantly increased cell proliferation in the ventricular zone (VZ), from which, in birds, the new cells migrate to the telencephalon, differentiate into neurons and settle in various brain regions [9,10], where they are recruited into functional circuits [11].

However, we recorded a decrease in total neuronal densities under ALAN exposure in the nidopallium caudale (NC), the medial striatum (MSt), and the HC, compared with controls [8]. The NC contains auditory relays [12], is involved in vocal communication, and in the integration of auditory information [13,14]; the MSt is part of the avian somatomotor basal ganglia [15] and is linked to visual perception and associative learning [16,17,18], and the HC processes spatial information [19,20] and plays a role in stress response [21]. In two of these regions, the NC and the MSt, the decrease in total neuronal densities was significant. This decrease in total neuronal densities, despite the increase in proliferation, suggests a net neuronal death. Taken together, the findings in our previous study [8] add to the notion of the deleterious effects of ALAN.

Two equally possible hypotheses might explain the results of our previous study, which indicated neuronal death despite the increase of cell proliferation in the VZ: (1) many of the new neurons produced in the VZ died under ALAN conditions, while migrating to their target destinations in the brain, hence only the relatively few that survived actually reached these target regions and had been recruited there. (2) ALAN causes a significant reduction in existing neurons, so that even increased proliferation and probable consequent increased influx of new neurons did not compensate for the loss due to neuronal death. Accordingly, our current work aims to help differentiate between these hypotheses, by investigating whether ALAN affects the recruitment of the new born neurons into their target regions in the brain. We exposed female zebra finches to ALAN for 7 weeks, and compared the recruitment of new neurons and total neuronal densities in selected brain regions (MSt, NC, HC) with control birds that were kept under dark nights. The ALAN intensity that we used was 5 lux, as it is ecologically relevant to birds [22,23] and had the greatest effect on cell proliferation in the VZ in our previous study [8]. In addition, in the present study, ALAN exposure was longer than in our previous one, to allow enough time for the new neurons to arrive at their final destination, and to check whether the previous exposure to ALAN was too short for the brain to reach an equilibrium between neuronal death and compensatory neuronal recruitment. We also measured plasma melatonin (MEL) concentrations as a key output of the central clock and which we previously showed was suppressed in response to a 3-week ALAN exposure in female zebra finches [8].

## 2. Results

### 2.1. ALAN Increases New Neuronal Recruitment in the MSt, HC, and NC

**Figure 1:**
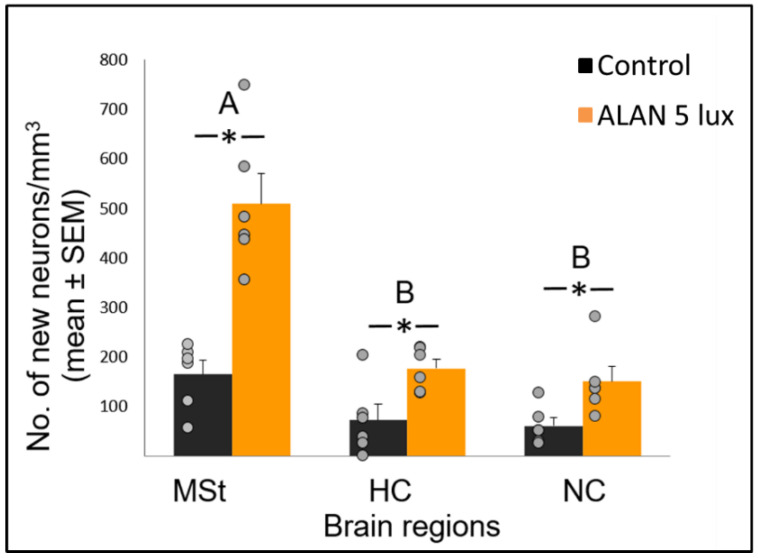
Neuronal recruitment (number of new neurons per mm^3^; mean ± SE) in three brain regions—medial striatum (MSt), hippocampus (HC) and nidopallium caudale (NC), of birds exposed to 5 lux ALAN and controls that remained in dark nights. Grey dots indicate individual data points, * indicates a significant difference between groups (*p* < 0.05), and different letters indicate significant differences between brain regions (*N* = 6 birds/group).

Exposure to 5 lux ALAN significantly increased the recruitment of new neurons, in analysis of the combined data for the three brain regions (F_(1,10)_ = 40.53, *p* < 0.0001; Figure 1). Recruitment was higher in the MSt compared with both the HC and the NC (F_(2,20)_ = 36.95, *p* < 0.0001), with marginally significant group x region interaction (F_(2,20)_ = 3.43, *p* = 0.052; “group” refers to Control/ALAN groups, “region” refers to MSt/HC/NC). Analysis of each brain region separately showed a significant increase of neuronal recruitment under ALAN in all the regions, the MSt, HC and NC (F_(1,10.8)_ = 39.59; *p* < 0.0001, F_(1,10.6)_ = 6.81; *p* = 0.025 and F_(1,9.6)_ = 8.61; *p* = 0.0156, respectively; Figure 1). There were no significant differences in neuronal recruitment among the five sections along the rostro-caudal axis and no significant interactions between section number and the other fixed variables (group and brain region).

### 2.2. ALAN Increases New Neuronal Recruitment in Sub-Regions of MSt at Different Rates

**Figure 2:**
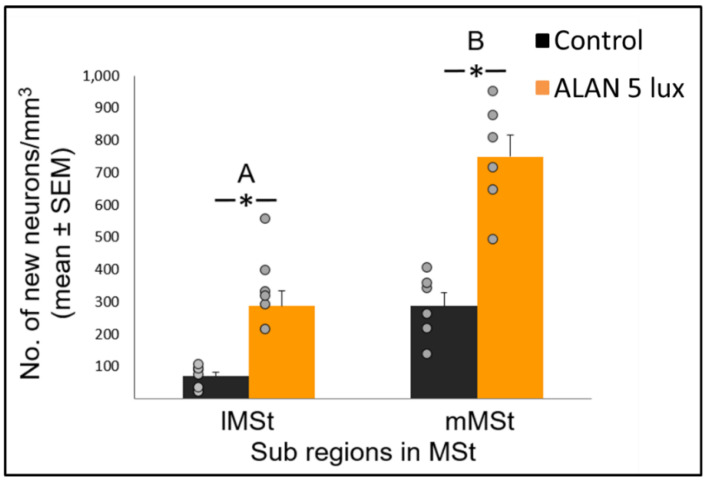
New neuronal recruitment (number of new neurons per mm^3^; mean ± SE) in the lateral (lMSt) and medial (mMSt) sub-regions of the medial striatum (MSt). Control birds were exposed to dark night whereas the experimental group was exposed to 5 lux ALAN. Grey dots indicate the individual data points, * indicates a significant difference between groups (*p* < 0.05), and different letters indicate significant difference between MSt sub-regions (*N* = 6 birds/group).

A further in depth analysis of the lateral (lMSt) and medial (mMSt) sub-regions of the MSt showed that ALAN significantly increased the number of new neurons recruited in both sub-regions (F_(1,10)_ = 45.16, *p* < 0.0001; Figure 2). In brains of birds that were exposed to ALAN, the number of new neurons in the lMSt was 3-fold higher than in the controls, whereas in the mMSt it was 2.5-fold higher than controls (F_(1,10.3)_ = 51.33, *p* < 0.0001 and F_(1,11.7)_ = 34.28, *p* < 0.0001, respectively; Figure 2).

Overall the number of new neurons recruited into the mMSt was higher than in the lMSt (F_(1,10)_ = 142.6, *p* < 0.0001; Figure 2) with no significant group x sub-region interaction. Also, there were not any significant differences in neuronal recruitment among the five sections along the rostro-caudal axis and no significant interactions between section number and the other fixed variables (group and MSt sub-region).

### 2.3. ALAN Increases Total Neuronal Densities in the HC

**Figure 3:**
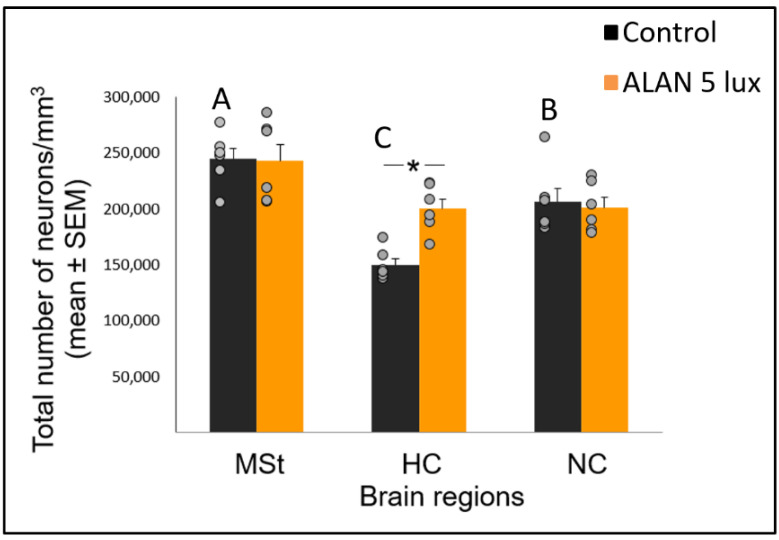
Total neuronal densities (number of neurons/mm^3^; mean ± SE) in the medial striatum (MSt), hippocampus (HC) and nidopallium caudale (NC) brain regions, in birds exposed to 5 lux ALAN, and controls that remained in dark nights. Grey dots indicate the individual data points, * indicates a significant difference between groups (*p* < 0.05), and different letters indicate significant differences among brain regions in the control group. (*N* = 6 birds/group).

Following our observation that ALAN increased the number of new neurons that were recruited into all the three brain regions, we also recorded total neuronal densities in these regions (i.e., BrdU labelled and unlabeled neurons). Overall, there was a significant difference among the regions (F_(2,14.5)_ = 47.56, *p* < 0.0001; Figure 3) and a significant group and brain region interaction (F_(2,14.5)_ = 6.17, *p* = 0.0115). Total neuronal densities increased in the ALAN group compared to control only in the HC (F_(1,10)_ = 9.638, *p* = 0.011), whereas no significant changes were observed in the MSt (F_(1,10)_ = 0.13, *p* = 0.91) and NC (F_(1,10)_ = 0.11, *p* = 0.75; Figure 3). In the control group, neuronal densities were significantly different between all the regions (F_(2,9.7)_ = 96.856, *p* < 0.0005).

### 2.4. ALAN Reduces Nocturnal Melatonin Levels

**Figure 4:**
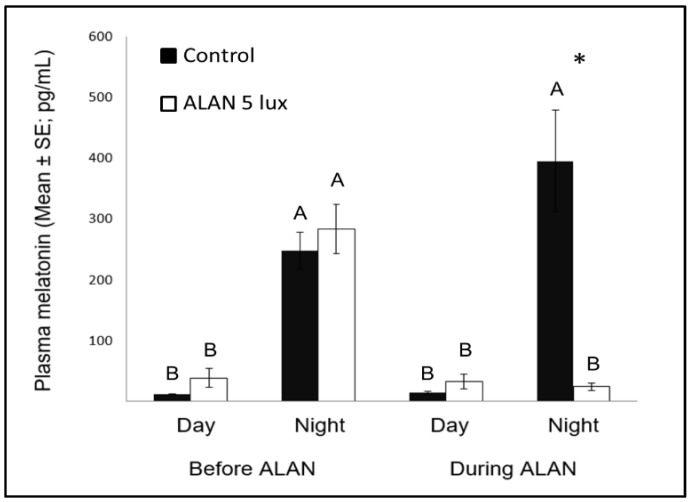
Day and night plasma melatonin levels (pg/mL; Mean ± SE) before and after exposure to 5 lux ALAN, compared with controls that remained under dark nights. * indicates significant difference between groups (*p* < 0.05) and different letters indicate significant differences between day and night (*N* = 6 birds/group).

We measured plasma MEL concentrations during midday and during the nights before and during exposure to ALAN. Overall, MEL levels were significantly affected by light-dark (LD) phase (F_(3,14.6)_ = 80.94, *p* < 0.0001) and the interaction between group and LD phase (F_(3,14.6)_ = 82.39, *p* < 0.0001). As expected, MEL levels were lower during the day than during the night before ALAN exposure, when both groups experienced complete dark nights (Figure 4). However, when the experimental group was exposed to 5 lux ALAN, nocturnal MEL levels were greatly reduced compared to control (F_(1,10)_ = 85.88, *p* < 0.0001; Figure 4).

### 2.5. ALAN Does Not Affect Body Mass

No significant differences were found in body mass between the ALAN and control groups, before and after exposure to ALAN (F_(1,10)_ = 0.587, *p* = 0.714). Mean body mass was 13.3 ± 0.35 g in the control group and 13.2 ± 0.32 g in the 5 lux ALAN group.

## 3. Discussion

### 3.1. ALAN Increases New Neuronal Recruitment in the MSt, HC, and NC

In various brain regions in birds, older neurons are constantly being replaced by new ones [10,24], and the assumption is that the total number of neurons within a brain region remains constant. However, in our previous study [8] we found, that despite an increase in cell proliferation in the VZ under ALAN conditions, total neuronal densities in target regions decreased. We suggested that this might be because ALAN impairs the brain’s ability to keep the total number of neurons within a certain region constant, due to death of the older, already-existing neurons in that region, or death of the new neurons, during their migration from the VZ and their recruitment into that region, or both. The present results, which show that new neuronal recruitment into the MSt, HC and NC was significantly increased (Figure 1), indicate that ALAN does not impair neuronal recruitment.

Neuronal recruitment was greater in the MSt compared to NC and HC in both control and ALAN groups (Figure 1). In addition, the increase under ALAN relative to control in the MSt was 3-fold, compared with 2.5-fold in the other two regions, with marginally significant (*p* = 0.052) group x region interaction, indicating a possible differential effect of ALAN on neuronal recruitment into different brain regions. The MSt is part of the somatomotor basal ganglia in birds, and also plays a role in avoidance behaviour [25], associative learning [26] and in visual perception [16,17,18]. Analysis of medial and lateral sub-regions of the MSt separately, revealed that ALAN increased neuronal recruitment in both (Figure 2). The mMSt receives dopaminergic inputs from the ventral tegmental region that is responsible for homeostatic and reflexive pathways [15,27,28], whereas the lMSt receives dopaminergic inputs from the substantia nigra pars compacta [29,30] and pallial input from regions involved in somatosensory, visual, auditory, and motor function [15,31,32]. Thus, our results indicate that ALAN affects both viscerolimbic and somatosensory functions. Under control conditions lMSt recruits fewer new neurons in comparison to mMSt (Figure 2). To the best of our knowledge this is novel information that has not been reported earlier. Similarly, in an earlier study, we observed that the survival of new neurons in the NC is influenced by their position within this region [33,34]. This finding led us then to suggest that when sampling a large region, questions about neuronal recruitment have to take into account also the spatial distribution of the new neurons within that region [35]. Our current findings in MSt support this suggestion. In addition, in our current study, exposure to ALAN significantly increased neuronal recruitment in both sub-regions of MSt, but it was more evident in lMSt than in mMSt. This differential increase suggests that mMSt is more resilient to ALAN than lMSt, which might be related to the functions of these sub-regions: if ALAN causes the birds to be more awake and active during the nights, this might affect lMSt, because it is connected to the substantia nigra that plays an important role in movement.

In the HC, which is involved in the processing of spatial information [19,20], the significant increase in the number of new neurons that were recruited under ALAN is contrary to the decrease that was found in Indian house crows [3]. However, these crows were acclimated, after being caught from the wild, only for a single week, shorter than the recommended three weeks to ensure thermogenic [36] and physiological [37] acclimation in birds. In addition, the crows were exposed to ALAN only for 10 days, and the marker that was used to detect new neurons was doublecortin, which is expressed in postmitotic migrating and still differentiating newborn neurons [38]. Therefore, it could be that the finding in crows represents a transient stage of young, not matured neurons, which have not been incorporated yet in their final destinations. The increase in the number of new neurons that we had found is also opposite to the decrease that was found in nocturnal mammals under ALAN conditions [39,40] and supports our suggestion that ALAN affects nocturnal and diurnal species differently [8]. It is unlikely that this increase was mediated by a greater demand for spatial memory, since both groups were acclimated to their cages for three weeks prior to the onset of the experiment, and were kept there throughout ALAN exposure, until they were killed. HC is also related to stress, however stress is known to decrease rather than increase neuronal recruitment, both in mammals [41] and birds [42]. Therefore, based on the current existing evidence in the literature, it is unlikely that stress can explain our observation of increased neuronal recruitment under ALAN. We therefore suggest that the increase in the HC might be a pathological response to ALAN, which changes the normal ratio between the influx of new neurons and death of others in this brain region. To test this possibility, in a currently ongoing study we also record apoptosis under ALAN conditions.

In the NC we also observed an increase in neuronal recruitment under ALAN conditions (Figure 1). NC is the centre for auditory relays and vocal communication [12], and because MEL receptors occur in high density in major brain components of the avian auditory and visual systems [43], it could be that there is a relation between the increased neuronal recruitment and the decrease in nocturnal MEL under ALAN (Figure 4). The low nocturnal MEL levels can facilitate higher activity during the night, a possibility that is supported by evidence of a negative correlation between MEL levels and locomotor activity in birds [44]. Moreover, locomotion activity is known to increase neuronal plasticity [45] and might also influence processing of auditory information. The possibility that our birds were more active during the nights under ALAN conditions might also result from sleep disruption, which occurs in birds that are exposed to light pollution [46]. We could not validate this hypothesis in our current experimental setup, but in a follow-up experiment we will also record nocturnal locomotion and sleep behaviour of control vs. ALAN-exposed birds.

### 3.2. A Possible Temporal Differential Effect of ALAN on Total Neuronal Densities in Various Brain Regions

In view that ALAN increased neuronal recruitment, as well as cell proliferation in the VZ, it remains to explain the reduction of total neuronal densities observed in our previous study [8]. It is possible that ALAN causes a significant reduction in existing neurons, so that even the increased proliferation and the probable consequent increased influx of new neurons could not compensate for the loss, due to greater neuronal death. We were aware that the 3-week exposure in our previous study might be too short to enable such compensation and retain constant neuronal densities. Therefore, in our present study we exposed birds to ALAN for seven weeks and found that MSt and NC maintained their total neuronal densities compared to controls. This suggests that the effect of ALAN is time-dependent, so that under longer ALAN exposure there is enough time for the brain to balance between the increase in new neurons (Figure 1 and Figure 2) and the death of older ones.

It is interesting to note that the temporal effect of ALAN on neuronal densities seems to be region-specific. This is because HC responded to ALAN differently than MSt and NC, both in the short-term [8], as well as in the long-term ALAN exposures (our present study). In the short-term (3 weeks), neuronal densities decreased in both the MSt and the NC, whereas no significant change was observed in the HC, similar to what had been found when Indian house crows were exposed to an even shorter ALAN duration (two weeks) [4]. However, in the long-term exposure of our current study (seven weeks), total neuronal densities in the HC increased compared to controls, whereas no changes were observed in MSt and NC. Thus, we suggest that while neuronal densities in the MSt and the NC are negatively affected during short-term ALAN exposure, these regions manage to compensate under long-term ALAN exposure. However, the HC exhibits an opposite pattern: during a short-term ALAN exposure neuronal densities are retained, but under long-term ALAN exposure they are significantly higher compared with control. If we assume that long-term ALAN exposure is the ecologically relevant duration, then it can be suggested that the differential temporal effect of ALAN on total neuronal densities renders some brain regions (as the MSt and NC) more resilient to it than others (as HC), because the former ones manage to retain their normal densities, whereas the density of the latter one departs from the normal level.

### 3.3. Possible Relation between Melatonin and Neuronal Recruitment in Birds

Nocturnal MEL levels under ALAN conditions were significantly lower compared to control birds that were exposed to dark nights (Figure 4), similar to previous reports in zebra finches [8,47], as well as in other bird species [23], diurnal mammals [48], and fish [49]. Evidently, very low ALAN intensities can suppress MEL biosynthesis not only in nocturnal [50], but also in diurnal species.

Another interesting comparison is the possible effect of MEL on neuronal survival in birds and other species. Our previous study [8] found that ALAN caused lower levels of nocturnal MEL, as well as decreased neuronal densities in some brain regions (MSt and NC), suggesting a possible relation between the two variables, which is in line with findings in mice, of a positive correlation between MEL and neuronal survival [51]. However, our present study does not support such correlation, because after a longer ALAN exposure neuronal densities in these regions were no longer different from those found in controls, although nocturnal MEL levels remained low. These different patterns between mice (which are nocturnal animals) and our birds (which are diurnal ones), supports our suggestion [8], that the physiological interpretation of ALAN might be different in nocturnal and diurnal animals.

### 3.4. Conclusions and Future Directions

Our study provides new evidence that ALAN increases new neuronal recruitment in the avian brain. We suggest that this increase might be a result of a compensatory response to neuronal death caused by ALAN, and/or due to increased locomotor activity during the night caused by sleep disruption. Our findings also indicate that ALAN has a differential temporal effect on various brain regions, some of them are more resilient and retain their normal numbers of neurons under long-term ALAN exposure, while others are more sensitive, and their neuronal densities are more affected. Further research is required to fully understand the effects of ALAN on neuronal plasticity in the avian brain, and therefore we intend to also record the effect of ALAN on apoptosis, and monitor the birds’ nocturnal locomotor activity and sleep. Finally, we plan to further investigate the intriguing indication for a possible temporal effect of ALAN on neuronal mechanisms in the brain, by studying these features in birds after a life-long ALAN exposure.

## 4. Materials and Methods

### 4.1. Experimental Design

**Figure 5:**
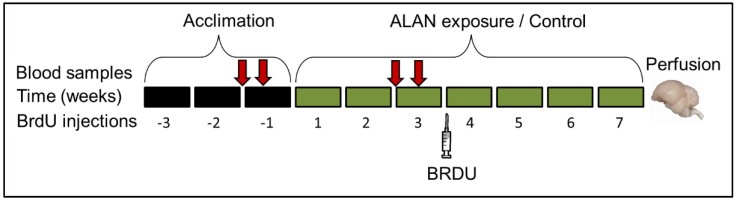
A schematic illustration of the experimental design. Top arrows indicate blood sampling; bottom syringe indicate BrdU treatment. See text for details.

A schematic description of the overall experimental design is presented in Figure 5. Adult female zebra finches were reared in outdoor breeding colonies at the Meier Segals Garden for Zoological Research at Tel-Aviv University, Israel. They were banded with numbered plastic rings for individual identification. At 90–200 days of age the experimental birds were removed from their native colonies and randomly allocated into two groups of six birds each (see below) in standard cages (67 × 33 × 33 cm). For a 3-week acclimation period, birds were kept under controlled LD cycle (14:10 h) and temperature (27 °C) that correspond to mean outdoor Israeli August conditions. These conditions were chosen because we previously found that neuronal recruitment was higher in birds that were kept under August compared with February conditions [52]. Birds were exposed to full spectrum white daylight fluorescent lamp (CFL, Hyundai, Skopje, Macedonia) emitting 1200 lux at perch level during daytime, and to complete darkness during the night. Following the acclimation period, birds were kept for additional 7 weeks under the same daytime (August) conditions, but during the night either under complete darkness (control group); or under 5 lux emitted by a full spectrum compact white fluorescent lamp (Hyundai, Beijing, China) at perch level (ALAN group). Spectra of all bulbs were measured by a spectrometer (Jaz spectrometer, Ocean Optics, Largo, FL, USA) and intensities by a digital light meter (TES-1337, TES, Taipei, Taiwan). Plasma MEL levels were measured during the last week of the acclimation period and during the third week of the experimental period. At the end of the third experimental week, birds were treated with bromodeoxyuridine (BrdU, SigmaUltra, Sigma-Aldrich^®^, St. Louis, MO, USA), a cell birth-date marker, to label new neurons in their brains, and four weeks later birds were killed and their brains were perfused, as described below. Food (millet seeds) and water were provided ad libitum during the entire study. As an indirect indication of the birds’ health, each bird was weighted six times every 15 days (three times prior to and three times during ALAN exposure). This study was approved by the Tel-Aviv University Institutional Animal Care and Use Committee (permit No. 04-17-011, issued on 5 March 2017) and was carried out in accordance with its regulations and guidelines regarding the care and use of animals for experimental procedures. All chemicals were purchased from Sigma, Israel, unless otherwise stated.

### 4.2. Melatonin Levels in Plasma

Four blood samples were taken from each bird (Figure 5): at midday on day 1 of week 3 of the acclimation and the experimental period, and at midnight 3 days following each of the midday samples. Blood collection at midnight was performed under a dim red light (lower than 0.1 lux) and with a black cloth pocket on the head of the bird after it was caught to avoid any light exposure. After drawing the blood, the bird was held in a temporary cage and the next bird was caught according to the above procedure. Blood sampling at night in the ALAN group was conducted under the same lighting conditions that were used for inducing ALAN (5 lux). Birds were sampled randomly. Blood was collected from the wing vein into microhematocrit-heparinized capillary tubes (Marienfeld, Lauda-Königshofen, Germany) and put immediately on ice. Plasma was separated by centrifugation for 8 min at 4000 rpm and kept at −80 °C until the analysis. Plasma MEL concentrations were measured by direct radioimmunoassay [53], which was validated also for zebra finches [8,54]. Specific MEL antiserum (Stockgrand Ltd., G/S/704-8483, University of Surrey, Guildford, UK) and tritium labelled MEL (*O*-methyl-3H) melatonin, TRK 798, specific activity: 3.07 TBq/mmol (83.0 Ci/mmol, Perkin Elmer, Waltham, MA, USA) were used. Sample radioactivity was measured in a scintillating β-counter for liquid samples (Packard Tri-Carb 2900 TR, Packard Instruments, Perkin Elmer). All samples were measured in a single assay. The intra-assay coefficient of variation was 4.3% and the assay sensitivity was 0.5 pg MEL per tube.

### 4.3. BrdU Administration, Histology, and Immunohistochemistry

**Figure 6:**
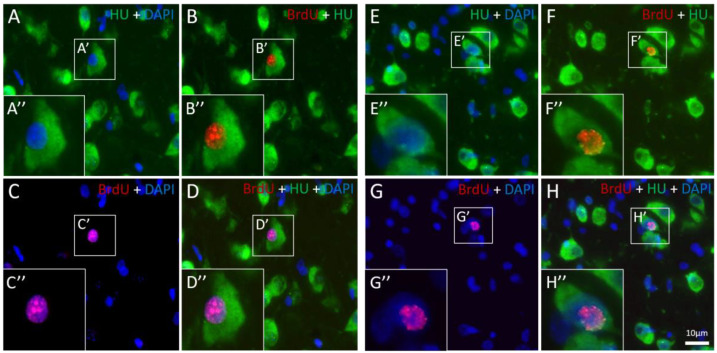
New neurons shown in microphotographs of the same field, (**A**–**D**) in the medial striatum (MSt); and (**E**–**H**) in the nidopallium caudale (NC) in the brain of a zebra finch (*Taeniopygia guttata*). HU staining (green) indicates neurons, BrdU staining (red) indicates nuclei of new neurons, and DAPI staining (blue) indicates cell nuclei. Overlapping of BrdU and DAPI staining is expressed as purple. Small frames (-′) indicate BrdU-HU co-labelled neurons, and big inserts (-″) are higher magnifications of these neurons. The scale of 10μm in **H** applies to **A**–**H**.

Four intramuscular injections of 130 μL (i.e., 100 mg/kg) BrdU were administered to each bird, a dose that is used to study neuronal recruitment in birds (e.g., [52]). Injections were administered on two consecutive days, at 9:00 and 15:00. The birds were left at the same experimental conditions for four more weeks. This protocol enabled labelling of the new neurons and their detection after they have completed their migration and were recruited at their target destinations in the brain. At the end of the experiment, the birds were weighed, killed and the brains were sectioned as described in [8]. Then, sections were processed for immunohistochemistry as described in [55]. Sections were incubated for 48 h at 4 °C, with primary antibodies: rat anti-BrdU IgG2a (diluted 1:200, Vector Labs, Burlingame, CA, USA) and neuronal specific antibody, anti-HuC/HuD mouse IgG2b, monoclonal 16A11 (diluted 1:200, Invitrogen, CarlsbaD, CA, USA). The secondary antibodies, F(ab)2 donkey anti-rat IgG-Cy3 (diluted 1:200, Jackson ImmunoResearch, West Grove, PA, USA), and Alexa Flour 488 donkey anti mouse IgG (H+L; diluted 1:200, Invitrogen), were applied at RT for 2 h. This staining protocol yielded neurons that were stained fluorescent green (with anti-Huc/HuD), and nuclei of new neurons that were stained fluorescent red (with anti-BrdU). Therefore, cells with colocalization of green cytoplasm and red fluorescent nuclei were identified as new neurons (Figure 6). Accordingly, we could record the location of BrdU^+^ neurons and count them in each section, as explained below.

### 4.4. Mapping and Quantification

**Figure 7:**
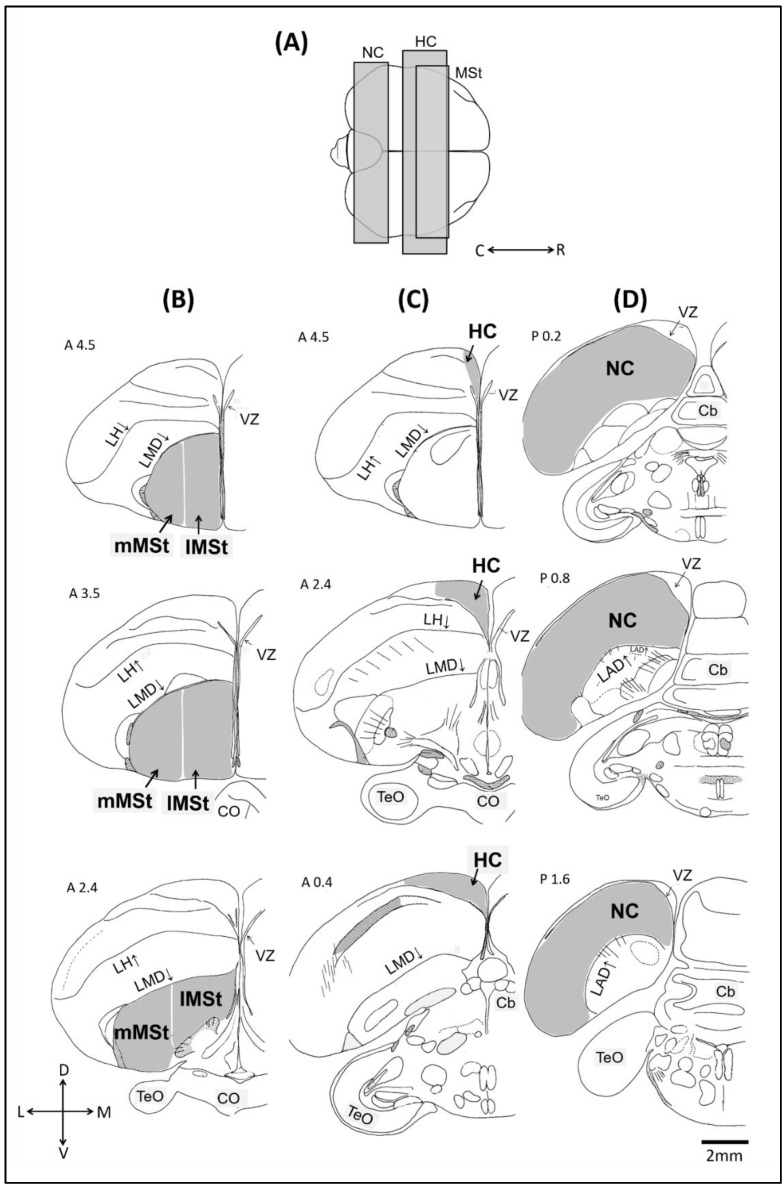
Schematic views of the three investigated brain regions. (**A**) Top view of the brain: rostral is to the right, and caudal is to the left. We indicate the range within which frontal sections were taken from the nidopallium caudale (NC), the hippocampus (HC) and the medial striatum (MSt). Five sections were sampled along the rostrocaudal axis of each brain region. For MSt (**B**), HC (**C**) and HC (**D**) only three are shown: the most rostral, the middle, and the most caudal (from top to bottom). Abbreviations: Cerebellum (Cb), Lamina arcopallialis dorsalis (LAD), Lateral ventricle (V), Tectum opticum (TeO). Orientations: Dorsal (D), Lateral (L), Ventral (V), and Medial (M).

We counted BrdU^+^ neurons in NC, HC, and MSt (see Figure 7A). To determine the boundaries of NC and HC, as well as the locations of the five sections that were sampled along the rostro-caudal axis in each of these brain regions, we followed our previous protocol as described in [56]. Those of MSt were determined according to [52] (Figure 7B–D). In addition, the MSt is known to consists of two functional sub-regions: the lateral portion of MSt (lMSt), and the medial portion of MSt (mMSt) (i.e., [15]). Therefore, in the MSt we mapped each sub-region separately. Because the boundary between these sub-regions is not very clear [15], we defined it as a straight line at an equal distance between the most lateral and most medial reach of MSt (Figure 7B).

We used a computerized brain-mapping system (Stereo Investigator, MicroBrightField, Williston, VT, USA; RRID: SciRes_000114) to draw the boundaries of the brain regions and sub-regions in each of the five sections that were sampled along the rostro-caudal axis of each region.

Within these boundaries, we determined the position and number of new neurons (co-labelling of BrdU-HU) and total neuronal densities (BrdU-HU labelled and HU labelled neurons). Mapping of each region and other measurements i.e., nuclear diameters of new and other neurons) were done as described earlier [52]. This information was used for the Abercrombie stereological correction [57] and enabled us to accurately estimate the number of BrdU^+^ neurons per mm^3^ in each brain region (for details see [52]).

### 4.5. Statistical Analysis

All analyses were performed using JMP^®^, Version 14 software, (SAS Institute Inc., Cary, NC, USA, www.sas.com). Data of neuronal recruitment were square-root transformed and those of MEL levels were log transformed to achieve normal distribution (Shapiro-Wilk test). Neuronal recruitment data were analyzed using a repeated measures full factorial mixed model analysis with section number and either brain region or sub-region as the within-subject fixed variables. Neuronal densities were analyzed by repeated measures mixed model analysis with brain region as the within-subject fixed variable. MEL levels were analyzed by repeated measures mixed model analysis with LD phase (day-before, night-before, day-during, and night- during) as the within-subject fixed variable. Group was the between-subject fixed variable, bird identification was the random variable and Toeplitz covariance structure was used in the mixed model analyses. Individual one-way analyses followed whenever a significant interaction was found. Tukey’s post-hoc test was conducted to determine specific differences. In all analyses the level of significance (α) was 0.05.

## Abbreviations

ALAN	Artificial light at night
HC	Hippocampus
MSt	Medial striatum
NC	Nidopallium caudale
NCL	Lateral caudal nidopallium
VZ	Ventricular zone
MEL	Melatonin
SE	Standard Error
lMSt	Lateral medial striatum
mMSt	Medial medial striatum
BrdU	Bromodeoxyuridine/5-bromo-2′-deoxyuridine
LD	Light-dark
IgG	Immunoglobulin G
CFL	Compact fluorescent lamp
DAPI	4′,6-diamidino-2-phenylindole
